# Assessing Cost-Effectiveness in Obesity (ACE-Obesity): an overview of the ACE approach, economic methods and cost results

**DOI:** 10.1186/1471-2458-9-419

**Published:** 2009-11-18

**Authors:** Rob Carter, Marj Moodie, Alison Markwick, Anne Magnus, Theo Vos, Boyd Swinburn, Michele M Haby

**Affiliations:** 1 Deakin Health Economics, Public Health Research Evaluation and Policy Cluster, Deakin University, 221 Burwood Highway, Burwood, Victoria 3125, Australia; 2 Public Health Branch, Department of Human Services, 50 Lonsdale Street, Melbourne Victoria, 3000, Australia; 3 Centre for Burden of Disease and Cost-Effectiveness, School of Population Health, The University of Queensland, Herston Road, Herston, Queensland, 4006, Australia; 4 WHO Collaborating Centre for Obesity Prevention, Deakin University, 221 Burwood Highway, Melbourne Victoria 3125, Australia

## Abstract

**Background:**

The aim of the ACE-Obesity study was to determine the economic credentials of interventions which aim to prevent unhealthy weight gain in children and adolescents. We have reported elsewhere on the modelled effectiveness of 13 obesity prevention interventions in children. In this paper, we report on the cost results and associated methods together with the innovative approach to priority setting that underpins the ACE-Obesity study.

**Methods:**

The Assessing Cost Effectiveness (ACE) approach combines technical rigour with 'due process' to facilitate evidence-based policy analysis. Technical rigour was achieved through use of standardised evaluation methods, a research team that assembles best available evidence and extensive uncertainty analysis. Cost estimates were based on pathway analysis, with resource usage estimated for the interventions and their 'current practice' comparator, as well as associated cost offsets. Due process was achieved through involvement of stakeholders, consensus decisions informed by briefing papers and 2^nd ^stage filter analysis that captures broader factors that influence policy judgements in addition to cost-effectiveness results. The 2^nd ^stage filters agreed by stakeholders were 'equity', 'strength of the evidence', 'feasibility of implementation', 'acceptability to stakeholders', 'sustainability' and 'potential for side-effects'.

**Results:**

The intervention costs varied considerably, both in absolute terms (from cost saving [6 interventions] to in excess of AUD50m per annum) and when expressed as a 'cost per child' estimate (from <AUD1.0 [reduction of TV advertising of high fat foods/high sugar drinks] to AUD31,553 [laparoscopic adjustable gastric banding for morbidly obese adolescents]). High costs per child reflected cost structure, target population and/or under-utilisation.

**Conclusion:**

The use of consistent methods enables valid comparison of potential intervention costs and cost-offsets for each of the interventions. ACE-Obesity informs policy-makers about cost-effectiveness, health impact, affordability and 2^nd ^stage filters for important options for preventing unhealthy weight gain in children. In related articles cost-effectiveness results and second stage filter considerations for each intervention assessed will be presented and analysed.

## Background

Obesity is now universally acknowledged as a major public health problem, both in children and adults [1,2]. This raises vital public health questions concerning what interventions are necessary to control the obesity epidemic; whether our early endeavours to confront obesity are the right choices; and whether they are sufficient to reverse the obesity trends.

Scarcity of funds dedicated to public health also means that difficult choices of what to fund to reverse the trend in unhealthy weight gain are inevitable. While there is nothing new about the task of making difficult choices in health care, policy-makers are now discussing with renewed interest the issue of how to set priorities which are evidence-based. Often choices have been driven by historical, political or commercial imperatives, but the importance of 'evidence-based policy' is increasingly being recognised [3]. Cost-effectiveness analyses can provide additional evidence-based information to help decision-makers set priorities and answer difficult questions such as those posed above. But for cost-effectiveness analysis to be taken seriously, economic analysts also need to address the broader concerns of decision-makers that go beyond simple formulae-based decision-making.

In recognition of the need for 'evidence-based' information to guide policy on obesity prevention, the Department of Human Services in Victoria, Australia, commissioned the Assessing Cost-Effectiveness in Obesity (ACE-Obesity) project in 2004. The aim of the project was to assist state and national policy-makers by providing best available modelled evidence on the effectiveness and cost-effectiveness of selected obesity prevention interventions, particularly amongst children and adolescents. ACE-Obesity followed earlier ACE studies in cancer [4], heart disease [5], and mental health [6,7], where the ACE approach was developed and successfully implemented.

The purpose of this paper is to overview the ACE approach to priority setting, together with the specific methods used in the ACE-Obesity study. In a companion paper [8], detail was provided on the methods used to calculate the likely impact of 13 obesity interventions on BMI, measured through changes in 'energy balance' and then conversion to anticipated health gain (using Disability Adjusted Life Years averted - DALYs). In this paper we complement that overview of health benefit results and methods, by providing an overview of cost results and key methodological issues associated with costing for the same 13 interventions. The bringing together of the benefits and costs is dealt with in a separate paper on the cost-effectiveness results (Moodie et al, 2009, as yet unpublished).

## The ACE Approach to Priority Setting

While the importance and need for priority setting in health care is generally accepted, the central question of how priority setting is to be achieved remains contested. There are a variety of approaches and models available, offered from a range of disciplines [9]. There are models developed by behavioural scientists based on achieving consensus; by epidemiologists/clinicians based on needs assessment; by philosophers based on notions of social justice; and by economists based on achieving efficiency.

A central issue is the extent to which priority setting approaches focus on 'technical analysis' or 'due process' for their legitimacy [9]. The 'technical school' is characterised by a reliance on rational decision rules, data sets and quantitative analysis. This school has in large part been the preserve of health economists (pursuing the goal of efficiency) and epidemiologists/clinicians (pursuing evidence-based guidelines and/or needs-based equity). Decisions are based on quantitative analysis and/or application of the correct rules, whether efficiency and/or equity focused. Provided one accepts these principles, results should give guidance to decision-makers on how services should be ranked.

In contrast, advocates of the 'due process' school question the assumption that it is possible to devise 'rational' decision rules and see the technical approaches as based on a simplistic view of the health care system. Here the task is less to refine the technical basis of decision-making, than to construct a process that enables proper debate and discussion to occur. This does not mean implicit rationing, but instead a system whereby decisions are made explicitly and the reasoning behind specific judgements is clearly explained.

The debate between the two schools, however, may be drawn too starkly in the literature [9-11]. There is no inherent conflict between action to provide more and better information on the costs, outcomes and evidence base for different interventions, and work to strengthen the processes for debating that information and arriving at judgements on priorities. The reality is that neither option alone is likely to fulfil the theoretical and practical requirements of an ideal approach to explicit priority setting. Both elements need to be involved in any approach to priority setting that is seeking strong theoretical foundations and empirical validity. The 'ACE' approach reflects our endeavours to develop such a joint approach.

On the technical side, the ACE methodology applies the key economic concepts of 'opportunity cost'; 'marginal analysis' and a 'clear concept of benefit' using standardised evaluation methods clearly documented in an evaluation protocol [9,12,13]. Undertaking the evaluations in this way as part of the priority setting exercise, addresses the reservations expressed by many economists about the simplistic use of league tables, where economic studies are assembled from the literature with little regard to differences in methods, context and setting [13]. The key characteristics of the ACE approach are:

• the rationale for the selection of interventions is clearly explained;

• the evaluation methods are standardised, documented and open to scrutiny;

• the setting, context and comparator is common to all interventions;

• country specific data are used, wherever possible, for demography, health system costs and disease incidence/prevalence patterns;

• information is assembled by a multi-disciplinary research team, preparing briefing papers to a standardised format agreed by a Working Group of stakeholders;

• a range of results is reported (around point estimates) reflecting explicitly the uncertainty of cost, process, outcome and value estimates; and

• the incremental cost effectiveness ratios are placed within a broader decision-making framework called '2^nd ^stage filter' analysis.

ACE Working Groups generally consist of stakeholders recruited from topic experts, clinicians and practitioners, relevant community organisations and policy-makers. The ACE approach aims to give these stakeholders a greater involvement in both the study design and conclusions, as recommended by the Panel on Cost Effectiveness in Health and Medicine [13]. The Working Group in ACE studies has an important role in achieving balance between the technical analyses and due process. On the technical side members contribute in areas of their expertise and discuss issues of method and evidence. On the 'due process' side, members ensure stakeholder interests and views are articulated; facilitate sensible interpretation of the technical analysis; assist with 'value' judgement aspects of the 2^nd ^filter analysis and help ensure transparency throughout the project.

## Methods

### The research question

The research question for the ACE-Obesity study was specified as:

What are the best options towards which state and national resources should be directed to reduce unhealthy weight gain in children and adolescents in Australia?

The reference year for all analyses was 2001, the latest year for which all key data sets were available.

### Study perspective

Given the nature of obesity interventions and their likely settings in community-based facilities such as schools and child care facilities, a broad 'societal perspective' was adopted, but with a major focus on the health sector implications. All known costs and outcomes of the interventions were identified and then either included or excluded in the measurement stage, with reasons clearly specified (such as availability of data or likelihood of being impacted by the intervention).

### Choice of comparator

One of the fundamental questions for economic evaluation is 'what difference the option for change makes to current policy?' Thus, the comparator to the interventions selected as options for change in the ACE studies was 'current practice' (refer Table 1). This recognises that resources currently being used could be integrated into a coordinated approach to the new intervention and/or that not all the benefits could automatically be attributed to the new intervention. This is often referred to as 'incremental analysis', with the resulting cost-effectiveness ratios referred to as incremental cost-effectiveness ratios (or 'ICERs'). In ACE-Obesity we modelled current practice as a 'no intervention' comparator as very little activity, if anything, was currently happening. Even where there was some minor activity (e.g. in schools), there was considerable uncertainty as to what constituted 'current practice', what it cost, and how effective it was. The associated disease management cost and health impacts of this 'no intervention' comparator were modelled through time and became the potential cost offsets and potential health gains for the new interventions.

**Table 1:** Evaluation design frame for interventions chosen

Interventions^1,2 ^and Setting	Target Population^3^	Cost Results			Key cost issues
		Gross Cost	Net Cost (Net Saving)	Cost Child^4^	
1. Active After School Communities Program [Child Care ^5^]. Runs 8 weeks in each of the 4 school terms.	Primary school children in Prep to Grade 6 (age 5-11 years). Number ≈ 99 000	$40.3 m [UI: $28.6 m-$56.2 m]^6^	$36.5 m [UI: $24.9 m-$52.6 m]	$407	i) Extensive & "lumpy" salary costs, particularly for regional physical activity co-ordinators; ii) sub-optimal capacity utilisation; iii) cost data modelled, not empirically-based; and iv) BMI outcomes not commensurate with high cost structure.

2. Multi-faceted program, including education to improve nutrition & increase physical activity, without an active physical education component [School-based].	Children in primary school Grades 1 and 2 (commencing in Grade 1, age 6 years). Number ≈ 114 630	$24.3 m [UI: $12.6 m-$39.2 m]	$9.0 m [UI: net saving of $9.1 m to net cost of $31.7 m]	$211	i) Costs over 2 year-period taken as representative 'annual cost' as reflects concomitant cohorts; ii) included central & school coordination costs for national program, but not teacher time (as integrated into curriculum); iii) assumed uptake by schools does not vary by type of school (public or private); and iv) parent involvement encouraged as part of program but not costed.

3. Multi-faceted program, including education to improve nutrition & increase physical activity, with an active physical education component [School-based].	Children in primary school Grades 1, 2 & 3 (commencing Grade 1, age 6 years). Number ≈ 114 630	$54.2 m [UI: $26.9 m-87.5 m)	($14.0 m) [UI: net saving of $41.9 m to net cost of $1.3 m]	$473	i) Costs over 3 year-period taken as representative 'annual cost' as reflects concomitant cohorts; ii) included central & school coordination costs for national program, but not teacher time (as integrated into curriculum); iii) physical activity component may pose problem for primary schools without specialist physical education teachers; iv) assumed uptake by schools does not vary by type of school; and v) parent involvement encouraged but not costed.

4. Multi-faceted program [School-based] targeted at overweight and obese children.	*Overweight or obese *children aged 7-10 years (Grades 2-5) at combined primary/secondary school. Number ≈ 17 000 over 4 years (4 200 each year)	$2.2 m [UI: $1.2 m to $4.1 m]	($1.2 m) [UI: net saving of $5.7 m to net cost of $0.38 m]	$129	i) Modelled as implemented over 4 years rather than implementing it to everyone eligible every 4 years; ii) involves a *peer-led *program using 8^th ^grade students, supported by counsellors, to help obese children in grades 2-5; iii) counsellors costed as publicly funded psychologists employed on part-time basis (different to trial).

5. Education program to reduce consumption of carbonated (fizzy) drinks [School-based].	Children in primary school 2 to 6 (age 7-11 years). Number ≈ 595 000 implemented over 5 years (119,000 each year)	$16.6 m [UI: $7.6 m -$32.2 m]	($26.7 m) [UI: net saving of $112.7 m to net cost of $32.0 m]	$28	i) Capacity calculated on seeing 1/5^th ^of schools each year, not all schools every year; ii) assumes each child receives intervention once during primary school; and iii) assumption of no additional school staff costs as sessions presented by trained project staff.

6. Education program to reduce TV viewing [School-based].	Children in primary school Grades 3 & 4 (age 8-10 years). Number ≈ 268 600	$27.7 m [UI: $12.7 m -$43.3 m]	($43.8 m) [UI: net saving of $81.8 m to net saving of $6.6 m]	$103	i) Modelling included national/state project officers to implement national program and full training costs for teachers, but not teacher time in the classroom (as integrated into curriculum); ii) 50% of schools participate in any one year; iii) assumed uptake by schools does not vary by type of school (public or private); and iv) parent involvement encouraged as part of program but not costed.

7. TravelSmart Schools [Schools/neighbourhoods & community organisations^7^]	Children in primary school Grades 5 & 6 (age 10-11 years). Number ≈ 267 700	$13.3 m [UI: $6.9 m -$22.8 m]	$12.58 m [UI: $6.1 m- $222.1 m]	$50	i) Large impact on cost-effectiveness from attributing a share of intervention costs to broader congestion, community & environmental objectives; ii) capacity utilisation not an issue for this intervention as 90% of costs are variable; and iii) cost data mostly modelled, not a strong empirical base.

8. Walking School Bus [Schools/neighbourhoods & community organisations].	Primary school children in Prep to Grade 2 (age 5-7 years). Number ≈ 7 840	$22.8 m [UI: $16.6 m-$30.9 m]	$22.53 m [UI: $16.35 m- $30.47 m]	$2908	i) Extensive set-up & overhead costs; ii) poor capacity utilisation; iii) attribution of costs to non obesity objectives; iv) empirical data coming from early developmental period of WSB program in Vic, Australia.

9. Reduction of TV advertising of high fat and/or high sugar foods & drinks to children [Media & marketing].	All Australian children aged 5-14 years. Number ≈ 2.4 million	$0.13 m [UI: $0.12 m-$0.14 m]	($299 m) [UI: net saving of between $133 m- $484 m]	$0.54	Key issue is exclusion of costs other than cost of monitoring/enforcing compliance with revised regulation. Excluded costs include: changing the regulations; any additional food costs to families in switch from non-core to core; impact on revenue stream of advertising companies & producers of non-core foods.

10. Family-based GP program targeted at overweight and moderately obese children [Primary care services ^g^]	*Overweight or moderately obese *children aged 5-9 years Number ≈ 9 685	$6.3 m [UI: $5.3 m-$7.4 m]	$2.95 m [UI: net saving of $1.06 m to net cost of $7.0 m]	$650	i) Intervention design includes costs of family participation, but no additional benefits are included from weight loss to family members other than the child; ii) potential for piggy-backing this intervention into other GP-based interventions; iii) low % of fixed costs means cost drivers for affordability (patient numbers), do not impact much on cost-effectiveness; and iv) majority of cost incurred by government, but cost impact on family still significant, with time costs a major factor.

11. Family-based targeted program for obese children [Primary care services^9 ^+ hospital setting delivery by multidisciplinary team]	*Obese *children aged 10-11 years. Number ≈ 5 800	$11.0 m [UI: $6.8 m-$18.3 m]	($4.0 m) [UI: net saving of $19.0 m to net cost of $2.4 m]	$1,896	i) Recruitment component adjusted from screening in schools in RCT, to opportunistic recruitment via GPs; ii) assumed 50% of 6,000 GPs already have calibrated scales and stadiometers necessary to measure weight; iii) 'intention to treat' approach adopted for costing (i.e. non completion still involved intervention costs), but full completion of visits required before benefits attributed.

12. Orlistat therapy for obese adolescents [Primary care services^9^].	*Obese *adolescents aged 12-16 years. Number ≈ 3 256	$6.3 m [UI: $1.4 m-$20.0 m]	$4.9 m [UI: $1.1 m-$15.9 m]	$1,935	i) Modelling incorporated opportunistic recruitment in Australian primary care setting (by GPs with dieticians providing dietary advice) and conservative adherence rates (65%); ii) only patients responsive to Orlistat assumed to continue past 2-week run-in period; iii) costs to parents in accompanying adolescent included; iv) high proportion of costs falling on patients/families (as Orlistat not on Pharmaceutical Benefits Schedule) impacts on access.

13. Laparoscopic adjustable gastric banding (LAGB) for morbidly obese adolescents [Hospital^10^].	*Severely obese *adolescents, aged 14-19 years, with private health insurance Number ≈ 4 120	$130 m [UI: $52 m-$265 m]	$53.4 m [UI: $20.1 m-$116.8 m]	$31,553	i) Definition of 'current practice'; ii) inclusion in costing of ongoing follow-up, including regular consultations and 2 LAGB replacements over lifetime; iii) cost data coming from early developmental period of LAGB (case series of 28 patients) extrapolated to eligible adolescent population; and iv) management of co-morbidities assumed to be same for intervention and comparator.

**Protocol issues common to all interventions**: i) Inclusion of time costs for adults/carers, but not children/adolescents; ii) exclusion of production gains/loses; iii) exclusion of unrelated costs in rest of life; iv) 'steady-state' costing, with program modeled in accordance with efficacy potential, assuming trained staff and infrastructure available; v) costs offsets based on mean reduction in BMI continuing over life of the child; vi) early set-up & development costs excluded (i.e. costs incurred before intervention commences, such as development of training packages); vii) annuitisation of capital, including human capital costs like training; and viii) full pathway costing, including recruitment and coordination.					

Notes:

^1 ^Current practice comparator defined as "no intervention" as programs either focussed on children previously inactive and/or minimal activity previously existed.

^2 ^The intervention period is defined as one representative year of "steady-state" operation, with "rest-of-life" modelling for all associated costs and benefits.

^3 ^Number of children participating in the intervention based on Australian population figures in 2001 and likely take-up rates. For some interventions, not all of the children/adolescents participating receive a health benefit from the intervention.

^4^Cost per child estimates do not include cost offsets.

^5 ^Includes child care centres, family day care and outside school hours care.

^6 ^95% uncertainty interval

^7 ^Includes State/Territory government, local government, community groups, recreation and sporting bodies, and private organisations.

^8 ^Current regulations limit adverts to 5 minutes every 30 minutes during 5 hrs of designated child slots & prohibits advertisements during 2.5 hrs per week of designated pre-school timeslots.

^9 ^Includes general medical practice (GPs), community health centres and other community-based and private-sector services.

^10 ^The hospital setting was not included in 'Healthy Weight 2008' [21], but this clinical intervention was included in the project for purposes of comparison and benchmarking.

### Target population

The actual Australian population of children and adolescents (aged 5-19 years) in the year 2001 was used and followed through time. The target groups within the Australian population for which the interventions were intended were clearly specified (refer Table 1). The relevant target group varied depending on the specific intervention; whether all persons in a particular age group, or a specific group of people (for example, all 8-11 year olds or overweight/obese 8-11 year olds).

### Time horizon

The time horizon for providing an intervention reflected its real-life application, but with costs and outcomes reported for the cohort of children eligible for a particular intervention in the representative baseline year 2001. The time horizon for the tracking of the costs and benefits arising from an intervention was the remaining life span of the target cohort, i.e. until death or age 100 years. A cohort multi-state life table approach was developed for this purpose [8].

Regardless of the time horizon chosen, all interventions were assumed to be in 'steady-state' operation, meaning that they were working at their full effectiveness potential and that trained personnel and/or infrastructure were available, and set-up costs were excluded.

### Discounting

Discounting at 3% per annum was applied to both costs and benefits. This rate approximates the long term bond rate, the rule of thumb often used in selecting the appropriate discount rate. It is also the rate recommended by a consensus panel of health economists in the USA for cost-effectiveness analysis [13].

### Selection of interventions

The guiding economic principle of opportunity cost -- that is, of benefit/benefit forgone in alternate uses of available funds -- rests on the careful selection and specification of current practice and options for change. The selection of interventions for analysis was a difficult and time consuming task, given the limited evidence of effectiveness and requirement for clear specification [8]. After discussion, the Working Group agreed to the following selection criteria:

1) relevance to current policy decision making;

2) availability of evidence of efficacy/effectiveness to support meaningful analyses, using a broad definition of evidence;

3) potential impact on addressing the problem of obesity;

4) the ability to specify the intervention in clear concrete terms to facilitate meaningful evaluation;

5) inclusion of a mix of interventions ranging from broad-based to narrower more specific interventions, and across a range of settings (community, schools, clinic, media); and

6) considerations of program logic.

The ACE-Obesity project had the resources over a two year period to evaluate the effectiveness and cost-effectiveness of 13 different obesity interventions in children and adolescents (Additional File 1). Priority was given to public health interventions that met 'relevance to current policy decision making' and 'availability of evidence to support the analyses'. Discussion around reasons for exclusion of other interventions can be found in Haby et al [8].

### Assessment of health benefits

DALYs saved over the child's lifetime was chosen as the measure of health gain. The methods for calculating the impact of the intervention on the Body Mass Index (BMI) post-intervention and then DALYs saved over the lifetime of the target population are described fully in Haby et al [8].

### Assessment of costs

A common convention in costing is to describe the analysis in three steps; identification, measurement and valuation [12].

#### Step One ~ Identification of costs

For the identification step, the societal perspective adopted means that costs to both public health care providers (Commonwealth, State and Territory governments) and private sector (clients/participants, their family/carers) were included, as well as costs to sectors other than health (for example, education and infrastructure).

The potential impacts of interventions on production in the general economy due to early return to work or reduced disease incidence (production losses/gains) were not included in ACE-Obesity, but are currently the basis of a separate study [14]. The inclusion of unrelated health care costs in additional years of life conferred by an intervention is a contentious issue amongst economists [12,13] and such costs were not included. While time costs for adults were included (e.g. parents and/or volunteers supporting an intervention), no allowance was made for the time costs of children and adolescents participating in an intervention.

#### Step Two ~ Measurement of costs

This step measures the quantities of resource used that stem from the intervention and its downstream effects.

##### (i) Intervention costs

Detailed pathway analysis was undertaken for each of the interventions. This entailed determining the probability of certain activities occurring and specifying the resources used in association with those activities. It was assumed that participants who did not adhere to the intervention incurred the intervention costs but received no benefit. One major issue of concern to accurate measurement was the attribution of costs to an intervention in situations where resources were jointly used by one or more programs. For example, in interventions in the school setting, the costs of teachers, materials and equipment may be shared by several programs. The criteria used to distribute such joint costs were tabulated and varied under sensitivity analysis so that users of the study results can satisfy themselves that they were reasonable.

##### (ii) Cost offsets

Cost offsets are savings in future health sector expenditure that can be attributed to the reduction in BMI due to an intervention. Health sector expenditure for obesity-related diseases, calculated by the Australian Institute of Health and Welfare, was used [15]. These disease specific estimates are satellite estimates of the main health expenditure estimates and are based on a national accounts approach. They include expenditure on health goods and services, health-related services and health-related investment. They do not include:

• expenditure that may have a 'health' outcome but which is undertaken outside the health sector, such as expenditure on building safer transport systems or the education of health professionals;

• expenditure on personal activities not directly related to maintaining or improving personal health;

• expenditure that does not have health as the main area of expected national benefit; or

• time costs.

It was assumed that the current (2001) average costs and clinical practice would be representative of future costs and practices.

To determine the reduction in obesity-related costs for a particular intervention, we used the same methods as used for the calculation of DALYs saved [8]. In summary, we first converted the total current costs for each obesity-related disease for each sex and 10-year age group, to rates by dividing by 2001 Australian population figures and applying these to each of the two 5-year age groups within the 10-year age group. Disease-specific rates were summed to give total cost rates for obesity-related diseases for each sex and 5-year age group. Total cost rates were extrapolated from 5-year to 1-year age groups for input into the base case life table using linear interpolation between data points. The change in the current and future BMI distribution of the target population due to the intervention was used to determine the potential impact fraction (PIF) for each 5-year age group and for males and females separately. The disease-specific cost rates for the intervention scenario were calculated as the base case cost rate x (1-PIF) for each age and sex category before input into the intervention life table. The difference between the base case and the intervention scenario gives the total obesity-related costs saved (cost-offsets). This difference was applied to the total population benefiting from the intervention to get total cost-offsets.

#### Step Three ~ Valuation of costs

In the valuation step, a unit price for each of the activities, together with the data source, was specified. Costs were measured in real prices for the reference year (2001). As interventions fell largely outside the health sector, adjustment was made using the relevant Consumer Price Index [16] rather than a health inflator. Intervention costs to the government health sector, clients/families, and non-health sectors were identified, measured and reported separately.

### Incremental cost effectiveness ratios (ICERs)

The cost-effectiveness ratios were determined as the incremental cost of the intervention divided by the incremental benefit and presented as cost (AUD) per DALY saved (both with and without cost offsets). In related articles the full cost-effectiveness results for each intervention assessed are presented and analysed in detail. The 'best options' are defined as interventions which are cost-effective as measured by the commonly used benchmark for cost-effectiveness in Australia of less than $50,000 per DALY (disability-adjusted life-year saved).

### Uncertainty testing and sensitivity analysis

Extensive 'uncertainty testing' was conducted to cover variation in those technical parameters (usually economic and epidemiological inputs) that have an impact on efficacy/effectiveness, unit costs, etc. 'Sensitivity testing', on the other hand, was defined to cover variation in key design features of an intervention, such as the delivery mode or the target age group and joint attribution of costs.

Simulation-modelling techniques (with Monte Carlo sampling) using @Risk software [17] were used to allow the presentation of the 95% uncertainty range around the benefits, costs and cost-effectiveness ratios. This approach is recommended by the Canadian Coordinating Office for Health Technology Assessment [18] and the US Consensus Panel on Cost-Effectiveness in Health and Medicine [13]. The probability distributions around the input variables were based on standard errors or the range of parameter values quoted in, or calculated from, the literature; and/or from expert advice on the likely scenarios under Australian conditions. The @RISK analysis also showed the input parameters with the greatest influence on the final results and hence provided an indication of research priorities if greater accuracy of results was desired.

### The 2nd Stage Filter Criteria

The 2nd stage filter analysis involved the assessment of issues that either influence the degree of confidence that can be placed in the cost-effectiveness ratios (such as the level of available evidence), or broader issues that need to be taken into account in decision-making about resource allocation. The filters used in the ACE-Obesity study were equity, strength of evidence, feasibility of implementation, acceptability to stakeholders, sustainability, and potential for side effects (Moodie et al 2009 as yet unpublished).

## Results

The costs for the 13 modelled interventions varied considerably, both in absolute terms and when expressed as a 'cost per child' estimate (refer Table 1). In terms of gross cost (i.e. ignoring potential cost offsets), costs for application Australia-wide, varied from AUD130m per annum (lap banding for severely obese adolescents), to less than AUD1m per annum (controlling TV advertising of 'junk food'; targeted multi-faceted school-based program), raising affordability as an important issue for policy-makers to consider. There were 3 interventions of high cost in Australian terms (i.e. >AUD40m per annum); 6 interventions of moderate cost (i.e. AUD10m-40m per annum; and 4 interventions of low cost (i.e. <AUD10m per annum).

When potential cost offsets were included, however, the results changed considerably for some interventions. One high cost intervention moved to a net saving (multi-faceted school-based with physical activity); three moderate cost interventions moved to a net saving (education to reduce carbonated drinks in schools; family-based targeted program for obese children; education program to reduce TV viewing); and two low cost interventions moved to a net saving (regulation of TV advertising; targeted multi-faceted school-based program). For those moderate to high cost interventions where potential cost offsets were small, this was because anticipated health impacts were small (as health impacts and cost offsets are correlated), and the combination of moderate to high costs with small health gains, meant achieving cost-effectiveness (i.e. less than AUD50,000 per DALY) was likely to be a challenge.

Expressing costs as a 'net cost per child' adds the important dimension of intervention reach and loading, to balance the simple focus on total costs. Some moderate to high cost interventions perform quite strongly on this indicator (Active After School Communities; TravelSmart; education to reduce TV viewing), as the setting and nature of the intervention mean that large numbers of children have access and/or capacity utilisation is stronger.

Detailed cost effectiveness and 2^nd ^stage filter results for each intervention will be published in upcoming papers.

## Discussion

The high variability in the cost results summarised in Table 1 raises affordability as a major policy consideration, in addition to concerns for effectiveness, efficiency and equity of access. In interpreting these cost results it is important to understand the impact of three key factors: first, the way in which the cost results are reported, which will suggest different take-home messages (i.e. 'gross cost', 'net cost' or 'cost per child'); second, the impact of intervention-specific factors which clearly vary between interventions (refer 'Key cost issues' column of Table 1); and third, the impact of the evaluation protocol which is common to all the interventions evaluated and affects each in much the same way (refer last row of Table 1).

Reporting costs as 'gross costs' (i.e. ignoring potential cost offsets) is important, because from a policy perspective, it closely approximates the financial budget that will be needed to operate the interventions. Reporting 'net costs' is important because cost offsets have a major influence on cost-effectiveness, as well as the ongoing budget implications of funding interventions over the longer term. The role of cost offsets will focus decision-makers on their achievability, as well as foster a concern for who receives those cost offsets. Reporting costs as a 'net cost per child' adds the important dimension of intervention reach and loading, which is a useful process indicator, but should not be confused with the higher order efficiency of achieving outcomes. Some interventions with low 'cost per child' results, for example, may still be ineffective in achieving BMI reductions (e.g. TravelSmart), while some with high 'cost per child' results could be very effective in achieving health gains (e.g. Lap Banding). Thus high 'costs per child' results reflect factors like total cost, targeting and capacity utilisation, but are not necessarily synonymous with poor cost-effectiveness.

Turning to the intervention-specific factors, it is clear that some interventions are over-burdened with extensive coordination and overhead costs, particularly when combined with poor utilisation of available capacity (e.g. Walking School Bus; Active After School Communities). As this situation applies to important current government initiatives at both the Commonwealth and State levels, we have undertaken careful examination of these cost structure and utilisation factors. The counterpoint to this observation is that some interventions have multiple objectives that include broader traffic congestion, community and environment goals (eg. TravelSmart, Walking School Bus). When a share of costs is attributed to these broader objectives, then cost performance is improved and/or affordability may be shared across a number of portfolios. Another important cost factor to consider is the potential to combine interventions or piggyback them on to existing programs (e.g. family-based GP program for moderately obese children).

In terms of the cost protocol, key points to note are the inclusion of time costs for adults; the assumption of 'steady-state' costing; the annuitisation of capital items (equivalent to a lease); and the approach to cost offsets. The 'steady-state' assumption means that the technical analyses did not include a detailed 'roll-out phase' and did not address implementation or 'learning curve' issues. This reflects the priority setting context of the analyses, where multiple interventions are being assessed, and the research question is framed in terms of the potential cost-effectiveness of interventions if they are implemented in accordance with their effectiveness potential. This does not mean, however, that practical issues related to the feasibility and acceptability of implementation are ignored - rather, they are considered in the 2^nd ^stage filter analysis. The 'steady-state' assumption keeps the technical analysis tractable and achieves comparability in the results across multiple interventions.

There are several limitations in our approach to the calculation of cost offsets. Firstly, the cost offsets are based on the mean reduction in BMI continuing over the life of child (bias towards over-estimation) [19,20]. The reported cost-offset results are dependent on this maintenance of benefit assumption. We have acknowledged that there is a potential danger for this assumption to over-estimate the cost-effectiveness results, but were constrained by the lack of evidence to the contrary in children. This will be addressed in individual papers where the sensitivity of results to this assumption will be explored.

In addition, cost offsets use a health sector rather than a societal perspective (bias towards under-estimation); time costs and other patient specific costs (eg travel costs) are not included in the health expenditure accounts; and not all health care expenditure could be allocated to specific diseases (around 13% involving ambulance assistance; aids and appliances; and aspects of community health), and as a result was not included (bias towards under-estimation). It is difficult to predict with any certainty the net effects of these biases.

Turning to the broader issue of the ACE approach to priority setting, the strengths of the ACE-Obesity study include an evidence-based approach; the use of a common economic protocol to ensure comparability of the results; extensive uncertainty and sensitivity testing; interpretation of cost-effectiveness ratios within a broader decision-making framework that includes consideration of second stage filter criteria; and the use of local data for demography, health system costs and offsets, disease incidence/prevalence, risk factors and disease burden [9].

The main limitations of the ACE-Obesity study relate to evidence of effectiveness which was discussed in detail in Haby et al[8] We use the Australian context to determine the health benefits, costs and incremental cost-effectiveness ratios. Therefore, care must be taken if trying to generalise the results to other countries. Some interventions may work differently in other countries due to differences in lifestyle, culture, beliefs and current practice in obesity prevention. The costs of implementing the interventions are also likely to vary between countries due to differences in salary structures, health systems, other unit costs and methods of implementation. The impact on total BMI units saved, DALYs averted and cost offsets will also vary according to differences in population size and structure and different disease rates between countries.

## Conclusion

Despite the current limitations in evidence, the ACE-Obesity study provides useful information for policy-makers. To our knowledge there have been no similar attempts at determining affordability and effectiveness of interventions across a wide range of obesity interventions in a comparable manner. Despite considerable uncertainty around key input variables, clear distinctions in cost between obesity interventions were found, together with logical explanations for the differentials. Nevertheless, until there is greater evidence, including better evaluations of new and current interventions for preventing overweight and obesity, many of our estimates should be considered provisional, though strongly indicative of the relative magnitude of the health gain and costs. For some interventions, particularly those which are currently funded, our work indicates areas where cost performance could be carefully studied, with a view to improvements in cost structure and capacity utilisation.

We hope the publication of individual intervention in future papers results will both encourage debate about future directions for health policy, and encourage further research to clarify those issues where current knowledge is lacking.

## Competing interests

The authors declare that they have no competing interests.

## Authors' contributions

RC and TV are the original developers of the ACE methodology. RC assumed primary responsibility for writing of the paper, whilst MM provided substantial input to the paper and developed the costing protocol for the ACE-Obesity project. MH project managed the project and made a significant contribution to the development of the economic methods. AMar, AMag and BS all inputted to the costing protocol. All authors have read and commented on the various drafts of the paper.

## Pre-publication history

The pre-publication history for this paper can be accessed here:

http://www.biomedcentral.com/1471-2458/9/419/prepub

## Supplementary Material

Additional file 1 **ACE-Obesity project: Description of interventions selected for evaluation**. Table provides a description of each of the thirteen interventions evaluated. Click here for file
