# Supplementary material for: Assessing Cost-Effectiveness in Obesity (ACE-Obesity): an overview of the ACE approach, economic methods and cost results
Source: BMC Public Health. 2009 Nov 18;9:419. doi: 10.1186/1471-2458-9-419 (PMC2785790; doi:10.1186/1471-2458-9-419)
Supplement: Additional file 1 — ACE-Obesity project: Description of interventions selected for evaluation. Table provides a description of each of the thirteen interventions evaluated. [file 1471-2458-9-419-S1.DOC]

**Additional File 1. ACE-Obesity project: Description of interventions selected for evaluation**

| **Intervention and setting** | **Description of intervention** |
| --- | --- |
| ***Child care*** | |
| 1. Active After School Communities program | The intervention is based on the Federally-funded Active After-School Communities program, which is currently being implemented by the Australian Sports Commission (ASC). It consists of: an invitation to primary schools and approved out-of school hours care services (OSHCS) to be involved in the program; preparation by schools/approved OSHCS of a physical activity needs analysis as part of their expression in the program; appointment of physical activity program coordinators for each OSHCS/schools; specification of a physical activity program for each OSHCS/school; delivery of the program (two or three times per week for one hour) in conjunction with local sporting clubs; supply of a nutritional afternoon tea to after school hours attendees. The duration of the program is eight weeks, and is available for four terms per year. |
| ***Schools*** | |
| 1. Multi-faceted school-based programme without an active Physical Education component | The intervention is based on the Know Your Body program (see intervention 3 below) but adapted for grade 1 Israeli children and did not include an active physical activity program (Tanir et al. *Prev Med* 1990;19:22-30). The regular teachers delivered the intervention over a two-year period and this consisted of 15 to 20 hours of teaching on health and nutrition, and physical activity per academic year for 2 years. Parental involvement was strongly encouraged through school events and educational booklets. |
| 1. Multi-faceted school-based programme with an active Physical Education component | The intervention is based on the “Know Your Body” (KYB) program developed by the American Health Foundation with the aim of reducing risk factors for cardiovascular disease through educational learning. The intervention is a modification of the KYB program that incorporates an active physical exercise component in addition to the education components (Manios et al. *Prev Med* 1999;28:149-159). The intervention was trialled on grade 1 children in a controlled (but not randomised) intervention trial in Greece. Regular classroom teachers delivered the intervention over a three-year period; the components included 13-17 hours of teaching on nutrition and health and 4-6 hours on physical fitness and activity per academic year; parental involvement with annual school meetings and information booklets; and two 45 minute practical physical exercise classes per week per academic year. |
| 1. Multi-faceted school-based program targeted at overweight and obese children | The intervention (based on a trial by Foster et al *J Consult Clin Psychol* 1985; 53: 538–540 provides for a peer-led program of counselling and social support for overweight or obese children in grades 2 to 5, run over 12 weeks. Peer counsellors (8^th^ grade students) are trained in 3 (1 hour) sessions to weigh children, check lunch boxes for nutritious foods and to recommend changes in eating and exercise habits. Each counsellor works with 3 or 4 students and meets with them 3 times weekly. Children are weighed weekly and rewarded with special stickers for weight loss of 0.23 kg or more. Participants are instructed for 15 mins each week by psychologists in behavioural techniques of record keeping, stimulus control, slowing eating, lifestyle activity and attitude change. They also attend a special weekly 15-min exercise class aimed towards non-competitive fun. Parents are invited to attend an introductory meeting and another in the tenth week where lessons in nutrition, physical exercise, positive reinforcement and behaviour modification are offered by psychologists. |
| 5. Education program to reduce consumption of carbonated (fizzy) drinks | This nutrition education intervention to reduce the consumption of sweetened carbonated beverages targets children aged 7-11 years. It was trialled in a randomised controlled trial in the United Kingdom (James et al. *Br Med J* 2004;328:1237-1243). It consists of four one-hour educational sessions (one session per school term) delivered by the study investigator with the assistance of the regular teachers. The sessions consists of information on achieving good health and promotion of drinking water. |
| 6. Education program to reduce TV viewing | The health promotion program to reduce TV viewing intervention was based on Bandura’s social cognitive theory and trialled in grade 3 and 4 children in a randomised controlled trial in the U.S.A (Robinson et al. *JAMA* 1999;282;1561-1567). Regular classroom teachers delivered the intervention in 18 hours of class time (over 6 months) comprising: 18 classes (30- 50 mins) conducted early in the academic year; a television turnoff challenge for 10 days; encouragement to follow a 7-hour a week TV budget thereafter aided by an electronic television time manager; additional lessons about intelligent TV viewing; final lessons in becoming an advocate for reducing media use; educational newsletters for the parents with strategies for limiting TV use. |
| ***Schools/neighbourhoods and community organisations*** | |
| 7. TravelSmart Schools | This program, targeted at children in years 5 & 6, was piloted in six schools in Victoria (DiPietro G and Hughes, <http://www.travelsmart.vic.gov.au/Web4/tsmart.nsf/>). It involves components designed to engage the whole school community (school councils, administrators, teachers, students, parents and families) including meetings and information sessions; a professional development program for teachers; approximately 20 hours over 4 weeks of classroom activities; activities and events designed to engage the whole school community (such as bike servicing and identification engraving by Police; ‘Leave the Car at Home’ Week); and promotion of the program within the local community. |
| 8. Walking School Bus | The intervention is based on the program as operated under the auspices of VicHealth(VicHealth <http://www.vichealth.vic.gov.au/Content.aspx>?). It is run by local councils and funded by Victorian Health Promotion Foundation to increase the number of primary school children walking to school . Children are accompanied by 2 adult ‘conductors’ (at a ratio of 1 adult to 8 children) and travel along a set route through a neighbourhood picking up children along the way at designated stops and delivering them to school. The volunteer conductors complete an induction program, are given police checks, and are covered by a Council’s volunteer insurance policy. |
| ***Media and marketing*** | |
| 9. Reduction of TV advertising of high fat and/or high sugar foods & drinks to children | The intervention precludes advertising of high sugar and/or high fat foods and beverages or fast food outlets during television viewing hours where a substantial proportion (15% or greater) of children up to the age of 14 years are in the viewing audience. The intervention will likely affect C, P and G programs shown between 7-8am and 3-9pm Monday to Friday and 6am to 1pm on Saturday and Sunday. This will require an extension of the existing regulatory framework in Australia, combined with the introduction of clearer definitions, to strengthen the monitoring and enforcement of the regulations. |
| ***Primary care services*** | |
| 10. Family-based GP program targeted at overweight and moderately obese children | The intervention is modelled on the LEAP (Live, Eat and Play) Study, a randomised controlled trial conducted by the Royal Children’s Hospital in Victoria in 2002-03 (McCallum et al. *J Paediatr Child Health* 2005; 41: 488–494). It consists of: recruitment through the Divisions of General Practice of participating GPs by letter and information/recruitment evenings; delivery of three 2.5 hour training sessions for participating GPs by a psychiatrist experienced in solution-focused family therapy; identification (from amongst their own patients) and recruitment by the GP of eligible overweight or moderately obese children and their parents; 4 individual consultations per patient (and parents) with the GP (first >40 minutes, then three shorter 20 - 40 minute visits over a 12 week period); use of brief solution-focused techniques that aim to identify and modify behavioural determinants of the child/family’s physical activity and nutrition. |
| 11. Family-based targeted program for obese children | The intervention is modelled on a Swedish study (Flodmark at al. Pediatrics 1993;91:880–884) consisting of: promotion of the intervention through the Divisions of General Practice; opportunistic recruitment of eligible overweight or moderately obese children and their parents by GPs from their own patients; medical examination and dietary counselling by a paediatrician and a dietitian; six family therapy sessions conducted jointly by a paediatrician and psychologist over a 14 to 18 month period, using brief solution-focused techniques, based on the ‘de Shazer’ method, to identify and modify behavioural determinants of the child/family’s physical activity and nutrition; three additional medical checks conducted through the course of the family therapy program. The intervention is based in the hospital setting, although it could be offered in an alternate setting. |
| 12. Orlistat therapy for obese adolescents | The intervention proposes treatment of obese adolescents with orlistat based on an American RCT (Chanoine et al. *JAMA* 2005; 293: 2873–2883). Orlistat (120mg) is given three times daily orally in conjunction with dietary, exercise and behavioural modifications, over a period of 12 months to adolescents aged 12-16 years, with a BMI of 2 units additional to the BMI units corresponding to the 95^th^ percentile of the age- and gender-specific BMI distribution. 17 GP and 4 dietician consultations are delivered to eligible adolescents under the existing primary care setting. |
| ***Hospital*** | |
| 13. Laparoscopic adjustable gastric banding for morbidly obese adolescents | Severely obese adolescents, aged 14-19 years with BMI ≥ 35 are eligible for the intervention (Fielding et al. *Surg Obes Relat Dis* 2005;1:399-405). It involves the laparoscopic surgical placement of a silicone prosthesis under general anaesthesia around the upper part of the stomach to produce a small stomach pouch. The main mechanism of action involves food gathering in the smaller pouch, which distends and gives a sensation of fullness with much smaller food portions and therefore decreases the appetite and calorie intake of the adolescent, resulting in weight loss. The intervention involves recruitment, pre-surgery consultations and investigations, surgery, and post surgery follow up and investigations. Currently LAGB is largely only available through private health insurance in Australia, and the intervention has been modeled on this basis. |
